# Supplementary material for: Clinical and Epidemiological Characteristics of Staphylococcus caprae Infections in Catalonia, Spain
Source: Microorganisms. 2025 Jan 1;13(1):53. doi: 10.3390/microorganisms13010053 (PMC11767556; doi:10.3390/microorganisms13010053)
Supplement: Supplementary file 1 [file microorganisms-13-00053-s001.zip › microorganisms-3385457-supplementary.pdf]

Supplementary Table S1. Diagnostic of 99 reported cases published between 1995-2023.

**Author citation**	**Country of origin.**	**Number of cases reported**	**Main diagnostic**	**Livestock exposure**	**Oxacillin susceptibility**	**Outcome**
Díez de los Ríos J et al. **2023** [1]	Spain	2	Infective endocarditis	Yes (2/2)	Susceptible (2/2)	Survived (1/2)
Vazquez O et al. **2023** [2]	Switzerland	1	Osteomyelitis toe phalanx	Unknown	Unknown	Survived
Domashenko P et al. **2023** [3]	Greece	1	Periprosthetic joint infection after hip arthroplasty	Unknown	Unknown	Survived
Scavelli K et al. **2022** [4]	USA	1	Endophthalmitis	Unknown	Unknown	Survived
Kinoshita Y et al. **2021** [5]	Japan	1	Heterotopic ossification after periprosthetic hip infection	Unknown	Unknown	Survived
Rodriguez Fernandez L et al. **2020** [6]	Spain	13	Skin and osteoarticular infections (n=11)<br>Bacteraemia (n=2)	Unknown	Unknown	Survived (13/13)
Fan Z et al. **2020** [7]	China	1	Lumbar spondylodiscitis	No	Susceptible	Survived
Hammami R et al. **2020** [8]	France	1	Infective endocarditis	Yes	Resistant	Survived
Rodríguez-Lucas C et al. **2019** [9]	Spain	1	Septic arthritis knee	Unknown	Susceptible	Survived
Ortega-Peña S et al. **2019** [10]	Mexico	1	Hip prosthesis infection	Unknown	Susceptible	Unknown
Gowda A et al. **2018** [11]	USA	1	Native vertebral discitis and psoas abscesses	Unknown	Unknown	Survived
Koo YJ. **2018** [12]	Korea	1	Puerperal sepsis	Unknown	Unknown	Survived
Hilliard CA et al. **2017** [13]	USA	1	Lumbar spondylodiscitis	Unknown	Susceptible	Died
Mazur E et al. **2017** [14]	Poland	1	Acute otitis media	Unknown	Unknown	Survived
Kwok TC et al. **2016** [15]	Scotland	1	Infective endocarditis	No	Susceptible	Survived
Pommepuy T et al. **2016** [16]	France	1	Hip prosthesis infection	Unknown	Susceptible	Survived
d'Ersu J et al. **2015** [17]	France	13	Bone and joint infections	No (0/13)	Unknown	Survived (13/13)
Seng P et al. **2014** [18]	France	25	Bone and joint infections	Yes (5/25)	Unknown	Survived (25/25)
Henry CR et al. **2014** [19]	USA	1	Endophthalmitis	Unknown	Unknown	Survived
Darrieutort-Laffite C et al. **2013** [20]	France	2	Bone and joint infections	Yes (2/2)	Susceptible (2/2)	Survived (2/2)
Kato J et al. **2010** [21]	Japan	1	Central line-associated bacteremia	No	Resistant	Survived
Kini GD et al. **2009** [22]	USA	1	Bacteraemia	No	Susceptible	Survived
Benedetti P et al. **2008** [23]	Italy	1	Meningitis	Unknown	Susceptible	Survived
Ross TL et al. **2005** [24]	USA	6	Bacteraemia	No	Resistant (6/6)	Survived (6/6)
Blanc V et al. **1999** [25]	France	1	Hip prosthesis infection	Unknown	Unknown	Survived
Spellerberg B et al. **1998** [26]	Germany	1	Bacteraemia	Unknown	Susceptible	Survived
Elsner HA et al. **1998** [27]	Germany	1	Intra-articular empyema knee	Unknown	Susceptible	Survived
Shuttleworth R et al. **1997** [28]	Canada	12	Bone and joint infections (n=10)<br>Mastoiditis (n=1)<br>Otitis externa (n=1)	No	Unknown	Survived (n=7)<br>Unknown (n=4)<br>Died (n=1; mastoiditis)
Vandenesch F et al. **1995** [29]	France	5	Bacteraemia (n=2)<br>Urinary tract infection (n=2)<br>Infective endocarditis (n=1)	No	Unknown	Survived (5/5)

References

1. Díez de los Ríos, J.; Hernández-Meneses, M.; Navarro, M.; Montserrat, S.; Perissinotti, A.; Miró J.M. *Staphylococcus caprae*: an emerging pathogen related to infective endocarditis. Clin. Microbiol. Infect. **2023**, 29(9), 1214–6. https://doi.org/10.1016/j.cmi.2023.06.006.
2. Vazquez, O.; De Marco, G.; Gavira, N.; Habre, C.; Bartucz, M.; Steiger, C.N.; et al. Subacute osteomyelitis due to *Staphylococcus caprae* in a teenager: A case report and review of the literature. World. J. Clin. Cases. **2023**, 16;11(20), 4897-4902. doi: 10.12998/wjcc.v11.i20.4897.
3. Domashenko, P.; Foukarakis, G.; Kenanidis, E.; Tsiridis, E. A Rare Case of *Staphylococcus caprae*-Caused Periprosthetic Joint Infection Following Total Hip Arthroplasty: A Literature Review and Antibiotic Treatment Algorithm Suggestion. Cureus. **2023**, 15, 1–7. https://doi.org/10.7759/cureus.39471.
4. Scavelli, K.; Priester, W.B.; Finn, A.P. Numerous White Retinal Lesions Following Cataract Surgery. JAMA Ophthalmol. **2022**, 140(10),1019-1020. doi: 10.1001/jamaophthalmol.2022.2152.
5. Kinoshita, Y.; Nakano, S.;Yoshioka, S.; Nakamura, M.; Goto, T.; Hamada, D.; et al. A Rare Case of Extremely Severe Heterotopic Ossification after Primary Total Hip Arthroplasty due to Persistent Mild Periprosthetic Joint Infection. Case. Rep. Orthop. **2021**, 2021,8849929. doi: 10.1155/2021/8849929.
6. Rodríguez Fernández, L.; Martín Guerra, J.M.; Dueñas Gutiérrez, C.J. Role of *Staphylococcus caprae* in nosocomial infection. Enferm. Infecc. Microbiol. Clin. **2020**, 38(9), 455–6. https://doi.org/10.1016/j.eimc.2020.01.022
7. Fan, Z.; Yang, Y.; Li, D.; Fei, Q. A rare lumbar pyogenic spondylodiscitis caused by *staphylococcus caprae* with initial misdiagnosis: case report and literature review. BMC. Surg. **2020**, 20(1), 200. doi: 10.1186/s12893-020-00860-2.
8. Hammami, R.; Ben Ali, Z.A.; Charfeddine, S.; Abid, L.; Kammoun, S. Endocardite infectieuse à *Staphylococcus caprae* compliquée de syndrome coronarien aigu [*Staphylococcus caprae* infective endocarditis complicated by acute coronary syndrome]. Med. Mal. Infect. **2020**, 50(6),531-533. doi: 10.1016/j.medmal.2020.04.008.
9. Rodríguez-Lucas, C.; Iriberri, I.; García-Arenzana, J.M.; Fernández, J. Septic arthritis caused by *Staphylococcus caprae* following arthroscopic meniscus tear repair in a patient without any foreign device. Enferm. Infecc. Microbiol. Clin

(English ed). **2019**, 37(6), 421–2. https://doi.org/10.1016/j.eimce.2018.10.013
10. Ortega-Peña, S.; Franco-Cendejas, R.; Salazar-Sáenz, B.; Rodríguez-Martínez, S.; Cancino-Díaz, M.E.; Cancino-Díaz, J.C. Prevalence and virulence factors of coagulase negative Staphylococcus causative of prosthetic joint infections in an orthopedic hospital of Mexico. Cir. Cir. **2019**, 87(4),428-435. doi: 10.24875/CIRU.19000690.
11. Gowda, A.; Pensiero, A.L.; Packer, C.D. *Staphylococcus caprae*: A Skin Commensal with Pathogenic Potential. Cureus. **2018**, 10(10), e3485. https://doi.org/10.7759/cureus.3485
12. Koo, Y.J. Puerperal septic shock and necrotizing fasciitis caused by *staphylococcus caprae* and *Escherichia coli*. Yeungnam. Univ. J. Med. **2018**, 35(2), 248-252. doi: 10.12701/yujm.2018.35.2.248.
13. Hilliard, C.A.; El Masri, J.; Goto M. *Staphylococcus caprae* bacteraemia and native bone infection complicated by therapeutic failure and elevated MIC: a case report. JMM. Case. Rep. **2017**, 4(9), e005112. doi: 10.1099/jmmcr.0.005112.
14. Mazur, E.; Żychowski, P.; Juda, M.; Korona-Głowniak, I.; Niedzielska, G.; Malm A.; et al. First report of a *staphylococcus caprae* isolated from middle ear fluid of an infant with recurrent acute otitis media. Ann. Agric. Environ. Med. **2017**, 24(3), 357–9. https://doi.org/10.5604/12321966.1233553.
15. Kwok, T.C.; Poyner, J.; Olson, E.; Henriksen, P.; Koch, O. *Staphylococcus caprae* native mitral valve infective endocarditis. JMM. Case. Rep. **2016**, 3(5), e005065. doi: 10.1099/jmmcr.0.005065.
16. Pommepuy, T.; Lons, A.; Benad, K.; Beltrand, E.; Senneville, E.; Migaud, H. Bilateral One-Stage Revision of Infected Total Hip Arthroplasties: Report of Two Cases and Management of Antibiotic Therapy. Case. Rep. Orthop. **2016**,2016,3621749. doi: 10.1155/2016/3621749.
17. D'Ersu, J.; Aubin, G.G.; Mercier, P.; Nicollet, P.; Bémer, P.; Corvec, S. Characterization of *staphylococcus caprae* clinical isolates involved in human bone and joint infections, compared with goat mastitis isolates. J. Clin. Microbiol. **2016**, 54 (1),106–13. https://doi.org/ 10.1128/JCM.01696-15
18. Seng, P.; Barbe, M.; Pinelli, P.O.; Gouriet, F.; Drancourt, M.; Minebois, A.; et al. *Staphylococcus caprae* bone and joint infections: A re-emerging infection?. Clin. Microbiol. Infect. **2014**, 20 (12), O1052–8. http://dx.doi.org/10.1111/1469-0691.12743

19. Henry, C.R.; Schwartz, S.G.; Flynn, H.W. Jr. Endophthalmitis following pars plana vitrectomy for vitreous floaters. Clin. Ophthalmol. **2014**,8,1649-53. doi: 10.2147/OPTH.S67855.
20. Darrieutort-Laffite, C.; André, V.; Leautez, S.; Tanguy, G.; Cormier, G. Arthrites septiques à Staphylococcus caprae [Staphylococcus caprae arthritis]. Med. Mal. Infect. **2013**, 43(3),131-2. doi: 10.1016/j.medmal.2013.01.003.
21. Kato, J.; Mori, T.; Sugita, K.; Murata, M.; Ono, Y.; Yamane, A.; et al. Central line-associated bacteremia caused by drug-resistant *Staphylococcus caprae* after chemotherapy for acute myelogenous leukemia. Int. J. Hematol. **2010**, 91(5), 912-3. doi: 10.1007/s12185-010-0568-y.
22. Kini, G.D.; Parris, A.R.; Tang, J.S. A Rare Presentation of Sepsis from *Staphylococcus caprae*. Open. Microbiol. J. **2009**, 3(1), 67–8. https://doi.org/10.2174/1874285800903010067.
23. Benedetti, P.; Pellizzer, G.; Furlan, F.; Nicolin, R.; Rassu, M.; Sefton, A. *Staphylococcus caprae* meningitis following intraspinal device infection. J. Med. Microbiol. **2008**, 57(Pt 7), 904-906. doi: 10.1099/jmm.0.2008/000356-0.
24. Ross, T.L.; Fuss, E.P.; Harrington, S.M.; Cai, M.; Perl, T.M.; Merz, W.G. Methicillin-resistant *Staphylococcus caprae* in a neonatal intensive care unit. J. Clin, Microbiol. **2005**, 43(1),363-7. doi: 10.1128/JCM.43.1.363-367.2005.
25. Blanc, V.; Picaud, J.; Legros, E.; Bes, M.; Etienne, J.; Moatti, D.; Raynaud, M.F. Infection sur prothèse totale de hanche à *Staphylococcus caprae*. Cas clinique et revue de la littérature [Infection after total hip replacement by *Staphylococcus caprae*. Case report and review of the literature]. Pathol. Biol (Paris). **1999**, 47(5), 409-13.
26. Spellerberg, B.; Steidel, K.; Lütticken, R.; Haase, G. Isolation of *Staphylococcus caprae* from blood cultures of a neonate with congenital heart disease. Eur. J. Clin. Microbiol. Infect. Dis. **1998**, 17(1), 61-2. doi: 10.1007/BF01584369.
27. Elsner, H.A.; Dahmen, G.P.; Laufs, R.; Mack, D. Intra-articular empyema due to *Staphylococcus caprae* following arthroscopic cruciate ligament repair. J. Infect. **1998**, 37(1), 66-7. doi: 10.1016/s0163-4453(98)90733-2.
28. Shuttleworth, R.; Behme, R.J.; McNabb, A.; Colby, W.D. Human isolates of *Staphylococcus caprae*: association with bone and joint infections. J. Clin. Microbiol. **1997**, 35(10), 2537-41. doi: 10.1128/jcm.35.10.2537-2541.1997.
29. Vandenesch, F.; Eykyn, S.J.; Bes, M.; Meugnier, H.; Fleurette, J.; Etienne, J.

Identification and ribotypes of *Staphylococcus caprae* isolates isolated as human pathogens and from goat milk. J. Clin. Microbiol. **1995**, 33(4), 888-92. doi: 10.1128/jcm.33.4.888-892.1995.
